# GCIP and SIRT6 cooperatively suppress *ITGAV* gene expression by modulating c-myc transcription ability

**DOI:** 10.1016/j.jbc.2025.108314

**Published:** 2025-02-13

**Authors:** Yi-Ching Huang, Tien-Ming Yuan, Bang-Hung Liu, Ruei-Yue Liang, Kai-Li Liu, Show-Mei Chuang

**Affiliations:** 1 Institute of Biomedical Sciences, National Chung Hsing University, Taichung, Taiwan; 2 Department of Surgery, Feng Yuan Hospital, Ministry of Health and Welfare, Taichung, Taiwan; 3 Department of Dental Technology and Materials Science, Central Taiwan University of Science and Technology, Taichung, Taiwan; 4 Department of Nutrition, Chung Shan Medical University, Taichung, Taiwan; 5 Department of Nutrition, Chung Shan Medical University Hospital, Taichung, Taiwan; 6 Department of Law, National Chung Hsing University, Taichung, Taiwan

**Keywords:** GCIP, SIRT6, c-myc, ITGAV, migration

## Abstract

Grap2 and CyclinD1 interacting protein (GCIP) has been suggested to function as a tumor suppressor and acts as a transcriptional regulator that negatively controls cancer cell growth, invasion, and migration. Knockdown of GCIP reportedly enhances cancer cell migration and invasion, but no previous study has examined the mechanism(s) by which GCIP suppresses migration/invasion in cancer cells. Here, we report that cDNA microarray-based expression profiling of A549 cells without and with knockdown of GCIP reveals that the expression levels of *ITGAV* and *ICAM-1* are negatively regulated by GCIP. *In vitro* co-immunoprecipitation and *in vivo* proximity ligation assays reveal that GCIP interacts with c-Myc. Sequence analyses reveal the presence of two c-Myc regulatory motifs (E-boxes) within the *ITGAV* promoter. Luciferase reporter and ChIP assays indicate that GCIP represses *ITGAV* transcription by interacting with c-Myc on the E-box binding sites of the *ITGAV* promoter region. Furthermore, GCIP interacts with SIRT6 *in vitro* and *in vivo* and cooperates with SIRT6, thereby linking its activity, to negatively regulate transcription at the E-box by modulating c-Myc transcription ability. Taken together, these findings contribute to our understanding of GCIP in tumorigenesis and identify a previously unrecognized function of GCIP: It can interact with c-Myc and SIRT6 at E-box binding sites of the *ITGAV* promoter region. Our data collectively reveal a regulatory network involving GCIP, SIRT6, c-Myc, and ITGAV, and suggest that the SIRT6-GCIP complex negatively regulates the oncogenic function of c-Myc in cell proliferation and migration.

The *c-Myc* proto-oncogene is a central regulator of cellular functions. It encodes c-Myc, which is a multifunctional transcription factor that plays important roles in regulating numerous cellular processes, including cell cycle progression, cell growth, differentiation, apoptosis, transformation, genomic instability, and angiogenesis. It is well known that monomeric c-Myc is not biologically functional; instead, it mediates transactivation in association with the related protein, Max, through an E-box motif (preferentially CACGTG) or a variant thereof to activate a diverse group of genes (1, 2). Dysregulation of the *c-Myc* protooncogene is one of the strongest activators of carcinogenesis, and overexpression of c-Myc is found in as many as 50% of all human tumors (3, 4, 5). Endogenous c-Myc is thought to bind to more than 5000 promoters in the human genome (6), including promoters and distal enhancers. Accumulating evidence indicates that transcriptional repression by c-Myc significantly promotes cell transformation, but the details underlying this process are unclear. Most researchers believe that transcriptional regulation by c-Myc is selective and that the interacting proteins play key roles in determining its molecular functions. c-Myc is reportedly recruited to core promoters through protein-protein interactions with acting transcription factors. In the most convincingly demonstrated mechanism for the repressive effect of c-Myc, it is reported to interact with Miz-1 on the transcriptional initiator (Inr) element to block the transcriptional activations of *p15* and *p21* (7, 8, 9). The c-Myc-Miz-1 interaction was predicted to account for 25%–40% of c-Myc-repressed genes (6), suggesting that there are additional interactors that contribute to c-Myc-mediated transcriptional repression. An example of such an interactor is NF-Y, which is bound by c-Myc to repress *pdgf-β* receptor transcription (10). The specificity of c-Myc-driven transcriptional responses likely is cell-, context-, and/or promoter-dependent. It remains to be demonstrated whether other mechanisms are involved in c-Myc-mediated gene repression. Moreover, the role of Max in the c-Myc-dependent repression mechanism is largely unexplored. It is also unclear how target gene specificity is achieved for c-Myc repression.

Numerous studies have examined the role of c-Myc in controlling tumor progression, but there is debate regarding its contribution to the propensity of a tumor for invasion and metastasis. Elevation in c-Myc expression has been found to downregulate α or β integrin expression in various cells, including keratinocytes, neuroblastoma cells, sarcoma cells, and hematopoietic stem cells (11, 12, 13, 14). Conversely, Liu *et al*. recently showed that c-Myc represses transcription from the promoters of the genes encoding αv and β3 integrin, which in turn reduces properties that are essential for metastasis (15).

Sirtuin 6 (SIRT6) is a mammalian homolog of the yeast deacetylase, Sir2 (Silent Information Regulator-2), which regulates fundamental processes in lifespan control, metabolism, and cancer biology (16). As a NAD-dependent protein deacetylase, SIRT6 deacetylates lysines 9 and 56 of histone H3 (H3K9 and H3K56) (17, 18, 19) to modulate HIF-1α- (20), NF-κB- (21), and c-Myc- (22) dependent pathways. Several studies have indicated that SIRT6 may play a tumor-suppressive role, attenuating and perhaps even antagonizing tumor development (22, 23, 24, 25). Notably, many of the molecular deficiencies that characterize SIRT6-knockout and -knockdown cells are also characteristic of cancer cells. In particular, genomic instability, imbalanced glucose homeostasis, aneuploidy, and overactive oncogenic signaling have emerged as hallmarks of many types of cancer (17, 20, 21, 22, 26). SIRT6 was also reported to join c-Myc in co-repressing the transcriptional activity of ribosomal genes. Loss of SIRT6 in mouse embryonic fibroblasts (MEFs) led to tumor formation independent of oncogene activation, and the tumors exhibited enhanced aerobic glycolysis (22). Furthermore, loss of SIRT6 in an *in vivo* model of colon cancer yielded a 3-fold increase in the number of adenomas, compared to those in control animals (22). SIRT6 expression was found to be downregulated in human pancreatic ductal adenocarcinoma and colorectal carcinomas compared to normal samples (22, 27). Finally, levels of SIRT6 were reportedly correlated with cancer progression and/or survival in colorectal cancer: Patients with low levels of nuclear SIRT6 had a shorter time to relapse and were more likely to relapse than those with high levels of nuclear SIRT6 (22). In addition, reduced SIRT6 expression seems to be correlated with the tumor initiation of liver and head and neck cancers (23, 28). Taken together, these studies demonstrate that SIRT6 functions as a tumor suppressor to inhibit the initiation and progression of cancers. However, the function of SIRT6 as a tumor suppressor is still controversial, especially in the context of different tumor types, contexts, and/or stages (29, 30).

Grap2 and CyclinD1 interacting protein (GCIP), an Id-like HLH protein that belongs to the class-V HLH proteins but lacks a basic DNA-binding domain, has been demonstrated to function as a dominant-negative transcriptional regulator of bHLH protein (31, 32, 33). Studies performed in mouse models *in vivo* and human cancer tissues suggested that GCIP may contribute to suppressing tumorigenesis in human cancers (34, 35, 36, 37, 38). However, relatively little is known about the molecular mechanisms underlying the ability of GCIP to suppress tumor progression. A few studies have provided molecular insights into the tumor-suppressive effects of GCIP. For example, GCIP has been shown to interact with cyclin D1 to attenuate the levels of Cdk4/cyclin D and Rb phosphorylation (32). Several human cancers frequently exhibit loss of heterozygosity (LOH) at chromosome locus 15q15, where GCIP is located (39). Ikushima *et al*. reported that GCIP physically interacts with oligodendrocyte transcription factor 1 (Oligo 1, a class-II bHLH transcription factor) to disrupt the TGF-β signaling-dependent association of Oligo 1 with Smad2/3 and that overexpression of GCIP suppresses TGF-β-induced cell growth and cell migration in glioma (40). Chen *et al*. showed that GCIP associates with Id1 to suppress tumorigenesis (41). Interestingly, Fujita *et al*. reported a novel mechanism wherein GCIP directly interacts with CBP to suppress CREB- and NF-kB-dependent transcription by inhibiting the interaction between CBP and RNA polymerase II (42).

Regardless of gender, lung cancer and gastric cancer are among the top five causes of cancer-related deaths (43). Small cell lung cancer (SCLC) comprises about 13% to 15% of lung cancer cases and has a 5-year survival rate of less than 7%. It is characterized by rapid proliferation, high vascularity, apoptotic imbalance, and early metastatic spread (44). Even after treatment, 60% of gastric cancer patients experience recurrence and liver metastasis (45). We previously showed that MEK/ERK signaling negatively regulates the stability of GCIP to attenuate its ability to suppress tumorigenesis (46). Accordingly, we sought to elucidate the mechanism by which GCIP suppresses tumor cell migration. In the current study, we report for the first time that GCIP downregulates the transcription of αv integrin (ITGAV, also called CD51, encoded by *ITGAV*) through specifically associating with c-Myc *in vitro* and *in vivo*. Through cDNA microarray analysis followed by ChIP assay, we further show that ITGAV is negatively regulated by GCIP, potentially *via* an interaction between GCIP and c-Myc on the promoter of the *ITGAV* gene. Max is essential for the interaction between GCIP and c-Myc. Moreover, GCIP associates with SIRT6, thereby linking its activity, through c-Myc binding, to repress the *ITGAV* promoter activity. These findings provide novel insight into the significance of GCIP in the aggressiveness of cancer and the associated mechanism. Moreover, these data define a previously unrecognized pathway for the negative regulation of c-Myc-mediated gene expression.

## Results

### GCIP regulates ITGAV and ICAM-1 gene expression

**Figure 1:**
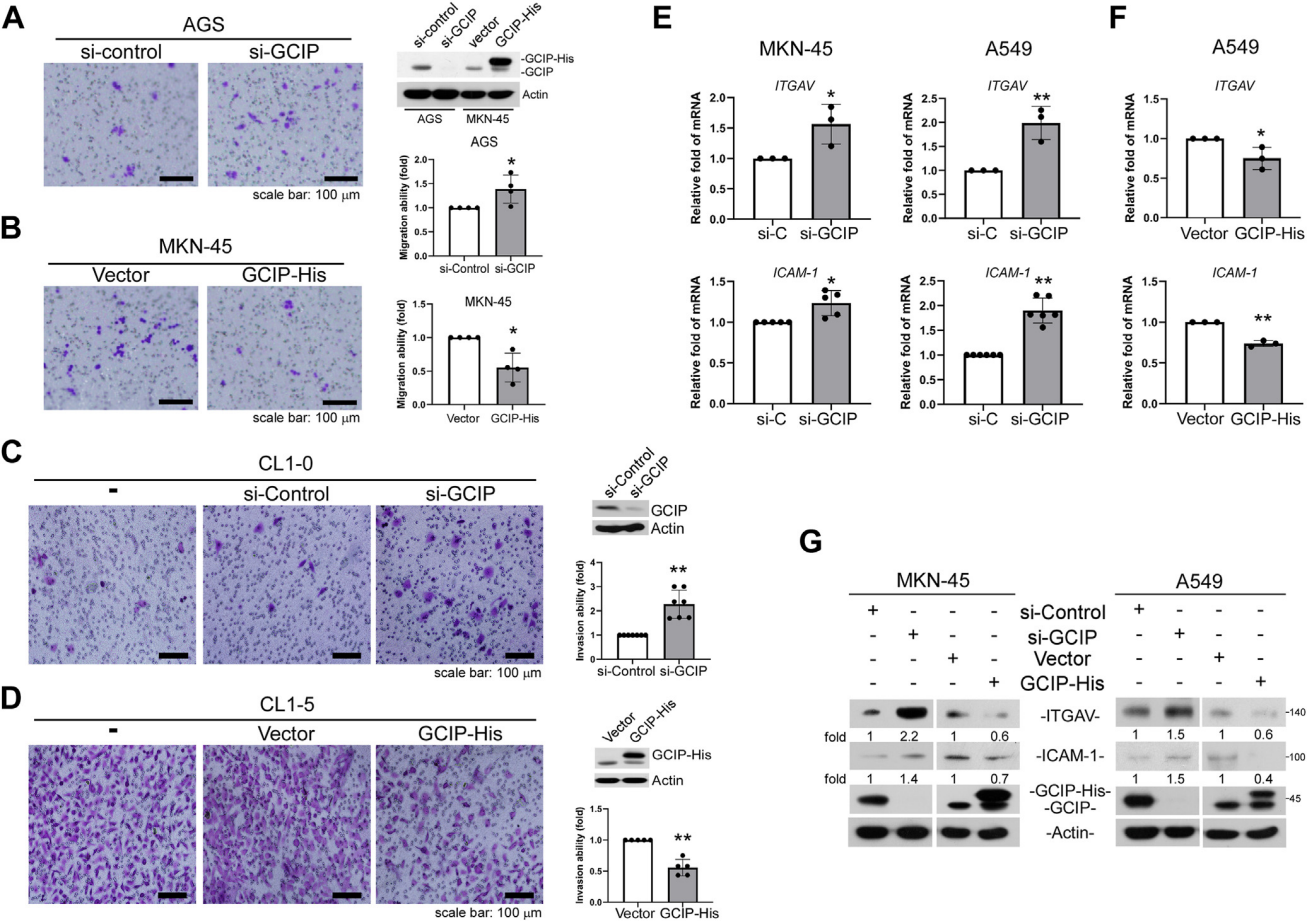
**GCIP negatively regulates cell migrativeness and invasiveness of human cancer cells**. *A*, *B*, the cell migrativeness of AGS and MKN-45 was evaluated by transwell migration assays. *C*, *D*, The cell invasiveness of CL1-0 and CL1-5 was evaluated by transwell invasion assays. *E*, cells were transfected with si-GCIP or (*F*) GCIP-His for 24 h, then *ITGAV* and *ICAM-1* expression were evaluated with qRT-PCR for mRNA expression. *G*, cells were transfected with si-GCIP or GCIP-His for 24 h, then ITGAV and ICAM-1 expression were evaluated with Western blot assays. Data are representative of four to six independent experiments. (∗*p* < 0.05, ∗∗*p* < 0.01, ∗∗∗*p* < 0.001).

In our previous studies, we demonstrated that GCIP inhibits cellular migration ability in human lung and gastric cancer; however, the regulatory mechanism underlying this effect remains unclear. First, we confirmed the tumor suppressor role of GCIP by manipulating its expression in human gastric and lung cancer cells. Consistent with our previous report (46), transwell migration assays indicated that ectopic GCIP attenuated cell migration in MKN-45 cells, whereas GCIP knockdown enhanced this parameter (Fig. 1, *A* and *B*). CL1-0 and CL1-5 are two representatives of a series of lung adenocarcinoma cell lines; a transwell invasion assay showed that the invasive ability of CL1-5 cells was 4- to 6-fold higher than that of CL1-0 cells, and a tracheal graft invasion assay revealed that CL1-5 cells exhibit invasiveness *in vivo* (47). Here, a cell invasion assay performed using a matrigel-coated transwell apparatus showed that GCIP knockdown enhanced the invasion of CL1-0 cells, whereas ectopic GCIP attenuated the invasion of CL1-5 cells (Fig. 1, *C* and *D*).

Next, we used an array approach to systematically identify GCIP-regulated genes that are involved in cell migration/invasion. A549 cells were transfected with siRNA specifically targeting GCIP for 24 h, and then cDNA microarray (Supporting information 1) analysis was performed, followed by KEGG pathway analysis of the differentially expressed genes. (Supporting information 2). A total of 858 genes were up-regulated and 171 genes were down-regulated in GCIP-knockdown cells compared to control cells (*p* < 0.05, fold-change ≧ 2). Among the upregulated genes, we chose 72 genes related to metastasis to further evaluate by qRT-PCR. We focused on *ITGAV*, which encodes integrin αv, an integrin family member known to be involved in cell adhesion and migration. *ITGAV* was previously reported to be down-regulated by c-Myc through the E-box region of its promoter (15). Here, it was found to be up-regulated by si-GCIP. We further examined the involvement of GCIP in ITGAV expression and whether c-Myc is critical for the GCIP-mediated suppression of ITGAV expression. We found that the mRNA levels of *ITGAV* and *ICAM-1 (the other gene reported by Liu et al*.*)* were significantly increased by the knockdown of GCIP in MKN-45 and A549 cells (Fig. 1*E*). Conversely, overexpression of GCIP attenuated the mRNA levels of *ITGAV* and *ICAM-1* (Fig. 1*F*). Immunoblot analysis confirmed that GCIP negatively regulates ITGAV and ICAM-1 protein expression, as these levels were increased by GCIP knockdown and decreased by GCIP overexpression in both MKN-45 and A549 cells (Fig. 1*G*). Thus, there appear to be negative correlations between GCIP and the expression levels of *ITGAV* and *ICAM-1*. We focused on ITGAV because it was significantly different in our cDNA microarray screening.

### GCIP interacts with c-myc

**Figure 2:**
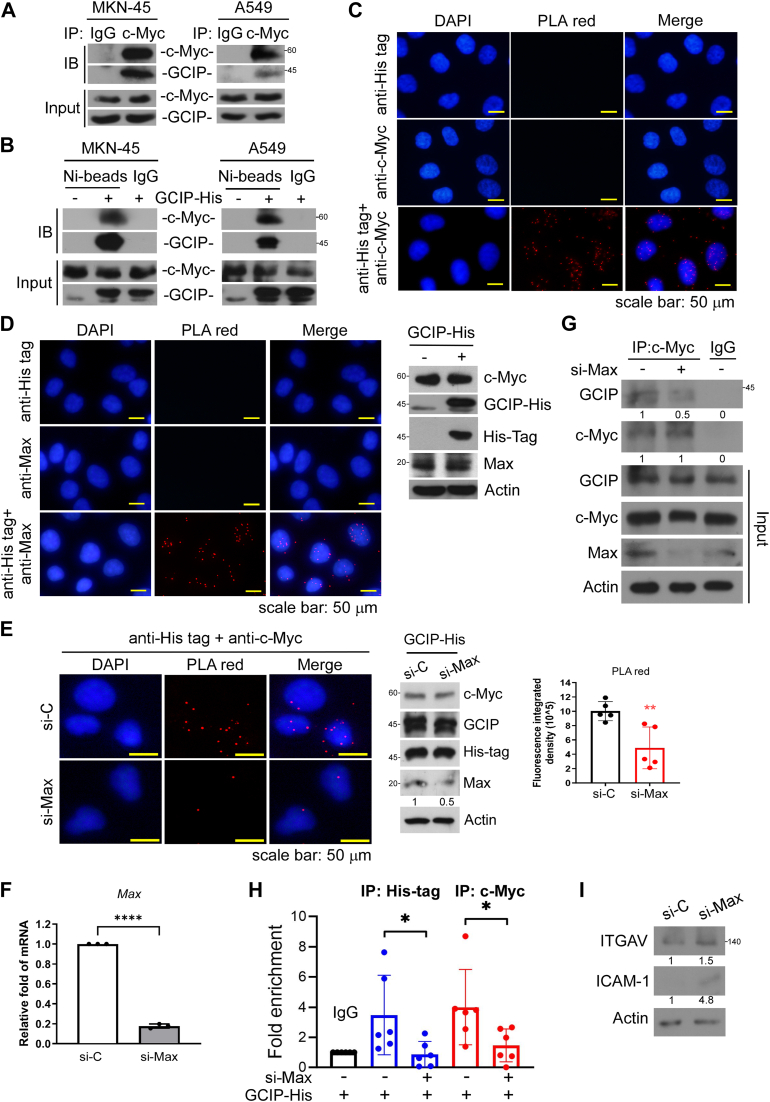
**GCIP interacts with c-Myc**. *A*, the endogenous c-Myc was immunoprecipitated followed by assaying the levels of associated GCIP with Western blot analysis. *B*, cells were expressed with His-tagged GCIP for 24 h followed by Nickle sepharose pull-down. The associated c-Myc were evaluated with Western blot analysis. *C*, A549 cells were transfected with GCIP-His for 24 h to perform PLA using anti-His tag and anti-c-Myc. *D*, A549 cells were transfected with GCIP-His for 24 h, then the interaction of GCIP with Max was determined by proximity ligation assays. *E*, A549 cells were co-transfected with GCIP-His and si-Max for 24 h, then the interaction of GCIP with c-Myc was determined by PLA. Data are representative of four to six independent experiments. *F*, The effect of si-Max was validated using qRT-PCR. *G*, A549 cells were transfected with si-Max for 24 h, and cell lysate was immunoprecipitated with c-Myc antibody followed by Western blot analyses. *H*, A549 cells were transfected with si-Max for 24 h before ChIP assays with anti-c-Myc or anti-His tag. Quantitative PCR analyses were performed on the immunoprecipitated DNA samples using primers specific for the E-box region in ITGAV gene promoter. *I*, A549 cells were transfected with si-Max for 24 h, and then ITGAV and ICAM-1 expression were evaluated with Western blot assays. Data are representative of three to eight independent experiments.

Given that GCIP is an Id-like HLH, we hypothesized that it could suppress *ITGAV* expression through a physical interaction with c-Myc. Indeed, immunoprecipitation assays revealed that c-Myc is associated with endogenous GCIP (Fig. 2*A*). A mutual interaction was also confirmed between ectopic GCIP-His and endogenous c-Myc (Fig. 2*B*). An *in situ* proximity ligation assay (PLA) further confirmed the biological relevance of the GCIP and c-Myc interaction *in vivo*. The distinct bright red fluorescence signal was observed markedly in the nucleus in the presence of antibodies against both GCIP and c-Myc, but not with anti-GCIP or anti-c-Myc alone (Fig. 2*C*), indicating that GCIP and c-Myc can be found in close proximity to one another and providing further support for their interaction. Together, our results indicate that GCIP interacts with c-Myc in cells.

It is well known that monomeric c-Myc is not biologically functional; instead, it must heterodimerize with Max to bind the E-box motif or a variant thereof to regulate a diverse group of genes. As an Id-like HLH transcription factor, GCIP cannot bind DNA directly. We therefore questioned whether Max could be involved in forming the GCIP-c-Myc complex. Indeed, our *in situ* PLA results indicated that GCIP and Max interact (Fig. 2*D*). Notably, the knockdown of Max attenuated the interaction between c-Myc and GCIP in PLA assays (Fig. 2, *E* and *F*). Co-immunoprecipitation and ChIP-qPCR also further confirmed that the occupancy of GCIP on the E-box of *ITGAV* was lost in the absence of Max (Fig. 2, *G* and *H*). In addition, the knockdown of Max increased the protein level of ITGAV (Fig. 2*I*), suggesting that Max might interact with c-Myc-GCIP to enable its complexation and then cooperatively inhibited the transcription of *ITGAV* and *ICAM-1*.

### GCIP interacts with c-myc on E-box binding sites

**Figure 3:**
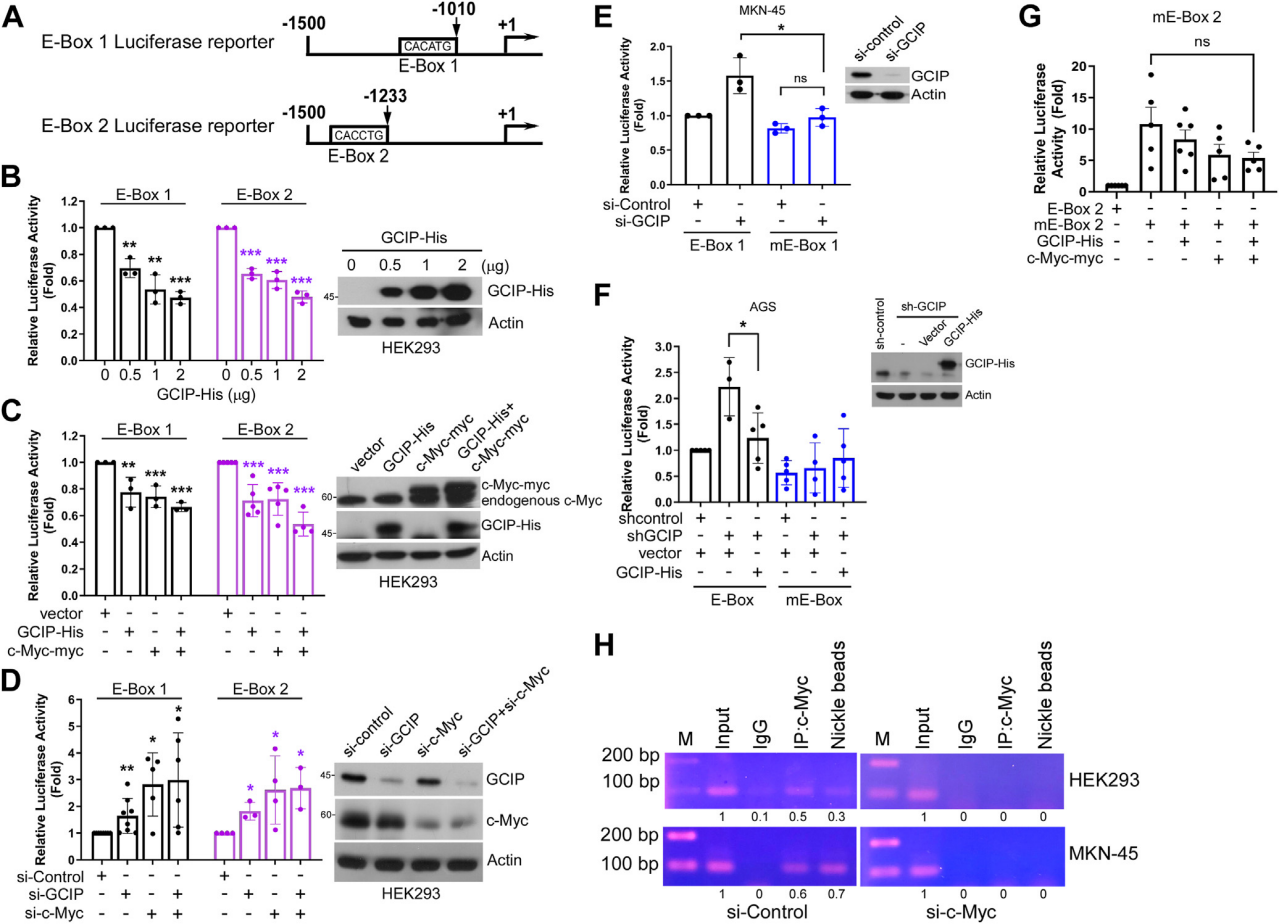
**GCIP interacts with c-Myc to downregulate the expression of the ITGAV gene through binding to its proximal promoters**. *A*, The schematic illustration of the E-BOX1 and E-BOX2 reporter construct upstream of ITGAV gene. *B*, GCIP-His was co-transfected with E-box 1-Luciferase or E-box 2-Luciferase reporter into HEK293. After 24 h, the luciferase activities were evaluated. *C*, E-box 1-Luciferase or E-box 2-Luciferase reporter was transfected into HEK293 with expression of GCIP-His, c-Myc-myc or co-expression of GCIP and c-Myc-myc. After 24 h, the luciferase activities were evaluated. *D*, E-box 1-Luciferase or E-box 2-Luciferase reporter was transfected into HEK293 with expression of si-GCIP, si-c-Myc or co-expression of si-GCIP and si-c-Myc. After 24 h, the luciferase activities were evaluated. *E*, Wildtype E-Box 1- or mutant E-box 1-Luciferase reporter was transfected into MKN-45 with expression of si-GCIP. After 24 h, the luciferase activities were evaluated. *F*, Wildtype E-box 1- or mutant E-box 1-Luciferase reporter was co-transfected with or without GCIP-His into AGS with expression of lentiviral-derived sh-RNA against GCIP. After 24 h, the luciferase activities were evaluated. The Western blot analyses (*B*–*F*) were representatives from one set of E-box reporter systems to indicate the transfection efficiency. *G*, mutant E-box 2-Luciferase reporter was transfected into HEK293 with expression of GCIP-His, c-Myc-myc, or co-expression of GCIP-His and c-Myc-myc. After 24 h, the luciferase activities were evaluated. The luciferase activity of E-Box 2 alone was calculated as 1-fold. *H*, MKN-45, and HEK293 cells were transfected with GCIP-His for 24 h before being assayed for chromatin immunoprecipitation analysis (ChIP) with anti-c-Myc or Nickel sepharose. PCR analyses were performed on the immunoprecipitated DNA samples using primers specific for the E-box region in ITGAV gene promoter reported by Liu *et al*. (15). A sample representing linear amplification of the total chromatin (Input) was included as control. Additional controls included non-specific immunoglobulins (IgG) as negative control. Results are expressed as the mean ± S.D. ∗*p* < 0.05, ∗∗*p* < 0.01, ∗∗∗*p* < 0.001 compared with controls from three to six independent experiments.

To assess whether the GCIP-mediated down-regulation of *ITGAV* expression occurred through the *ITGAV* gene promoter, we cloned the E-box (CACATG) region of the *ITGAV* gene promoter into a luciferase reporter vector and performed luciferase reporter assays (15) (Fig. 3*A*, E-box 1). A second reporter construct was designed to include a second putative E-box sequence (CACCTG) that was identified following the CACATG element (Fig. 3*A*, E-box 2). The relative luciferase activities of E-box 1 and E-box 2 gradually decreased as the ectopic expression of GCIP increased (Fig. 3*B*). Consistent with the findings of Liu *et al*. (15), c-Myc repressed the luciferase activity of the generated construct. This luciferase activity was further suppressed by co-expression of GCIP and c-Myc (*C*), and enhanced by knockdown of GCIP or c-Myc (Fig. 3*D*). As expected, mutation of the E-boxes abolished the GCIP- or c-Myc-mediated repression of ITGAV promoter activity (Fig. 3, *E*–*G*), providing further evidence that GCIP directly represses ITGAV transcription. ChIP-PCR analysis revealed significant occupancy of the E-box site by anti-c-Myc and -His antibodies, but not control non-specific IgG. The occupancy of c-Myc on the E-box was observed with or without ectopic GCIP expression (Fig. 3*H*), suggesting that GCIP did not hinder c-Myc from binding to the E-box regions. However, GCIP did not localize on the E-box in the absence of c-Myc (Fig. 3*H*, right panel), indicating that c-Myc is required for GCIP to occupy the E-box regions. The co-occupancy of the ITGAV gene promoter by c-Myc and GCIP suggests that these two proteins may interact to coordinate ITGAV expression. Together, these findings indicate that GCIP may negatively regulate ITGAV expression through interaction with c-Myc on the E-box regions.

### GCIP coordinates SIRT6 to bind c-myc

**Figure 4:**
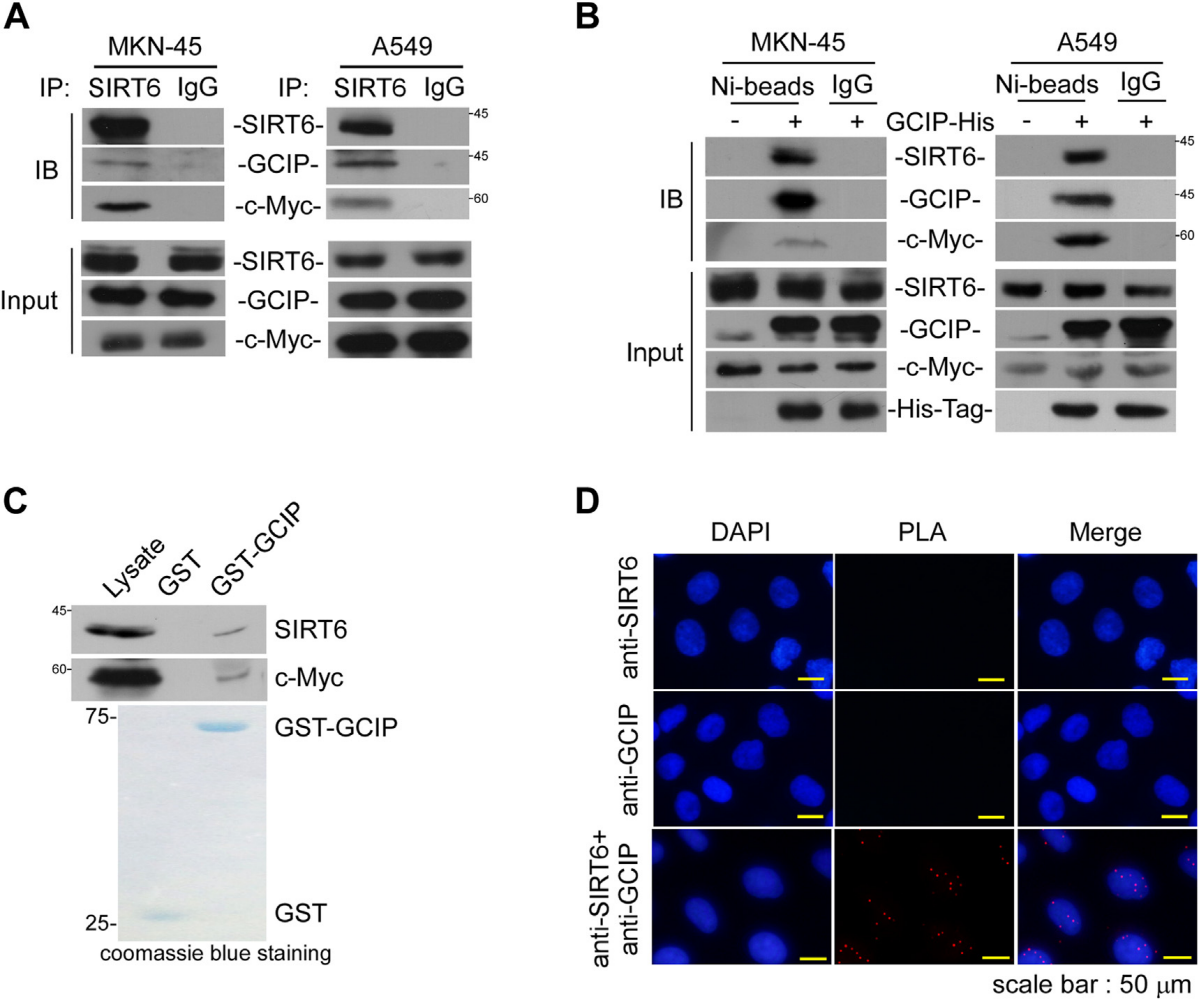
**SIRT6 associates with GCIP and c-Myc**. *A*, cell lysates were prepared to perform immunoprecipitation using anti-SIRT6. The associated GCIP and c-Myc were then evaluated by Western blot analysis. *B*, cells were transfected with GCIP-His for 24 h and then the cell lysates were prepared to perform Nickle beads pull-down followed by Western blot analysis using antibodies against SIRT6, c-Myc, and GCIP. *C*, the cell lysates were pulled down by recombinant GST-GCIP *in vitro* followed by Western blot analysis to detect the associated SIRT6 and c-Myc. *D*, A549 cells were transfected with GCIP-His for 24 h to perform PLA using anti-His tag and anti-SIRT6. Data are representative of three to six independent experiments.

As SIRT6 is an interacting partner of GCIP (36) and reportedly regulates the expression levels of a subset of c-Myc-controlled genes (22), we questioned whether SIRT6 is present in the GCIP-c-Myc complex. Indeed, our co-immunoprecipitation assay suggested that GCIP interacts with SIRT6 and c-Myc, and *vice versa*, in MKN-45 and A549 cells (Fig. 4, *A* and *B*). An *in vitro* pull-down assay also indicated that SIRT6 and c-Myc were detected in the GCIP-pulldown complex (Fig. 4*C*). *In situ* PLA provided further evidence for an interaction between SIRT6 and GCIP within cells: The distinct bright red fluorescence signal was observed markedly in the cytosol in the presence of antibodies against SIRT6 and GCIP, but not with anti-SIRT6 or anti-His tag alone (Fig. 4*D*), indicating that SIRT6 and GCIP can be found in close proximity to one another.

**Figure 5:**
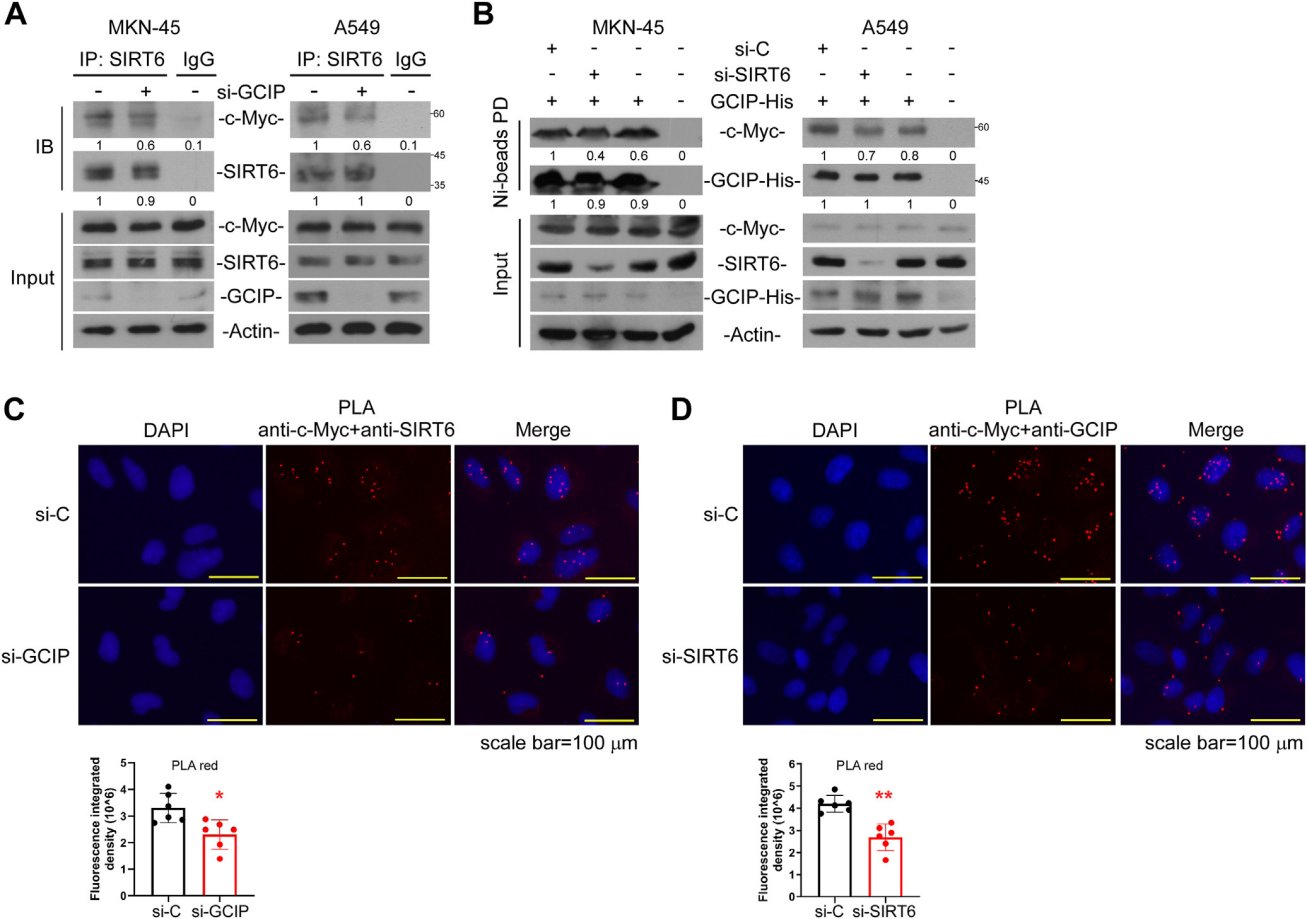
**Cooperative modulation of GCIP and SIRT6 on c-Myc complex.***A* and *B*, Cells were transfected with si-GCIP or si-SIRT6 for 24 h to perform coimmunoprecipitation using anti-SIRT6 or Nickle-sepharose beads pull-down. *C* and *D*, cells were transfected with si-GCIP or si-SIRT6 for 24 h to perform PLA. Data are representative of five to six independent experiments.

To further assess the role of GCIP in the association of SIRT6 with c-Myc, we knocked down GCIP and then performed coimmunoprecipitation assays with anti-c-Myc or anti-SIRT6. Knockdown of GCIP slightly decreased the interaction of SIRT6 with c-Myc compared to the control in both cell lines (Fig. 5*A*). Notably, the knockdown of SIRT6 also slightly attenuated the interaction between GCIP and c-Myc (Fig. 5*B*). PLA experiments further suggested that both GCIP and SIRT6 contributed to the interaction with c-Myc (Fig. 5, *C* and *D*). These results suggest that the interaction between GCIP and SIRT6 might facilitate the formation of the c-Myc transcriptional complex on inhibitory binding regions, such as that found at the ITGAV promoter.

### GCIP cooperates with SIRT6 to repress the ITGAV promoter through c-myc

**Figure 6:**
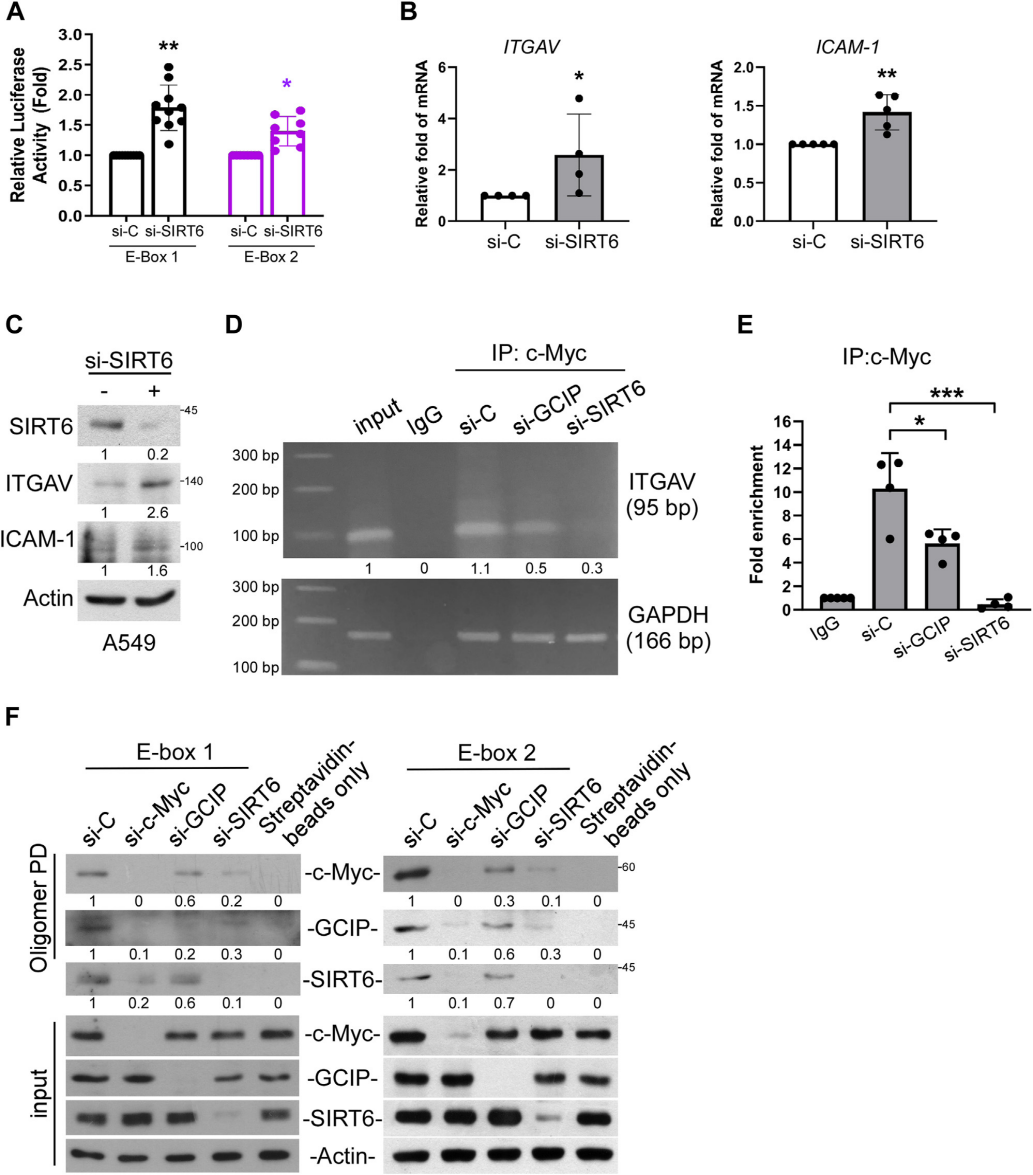
**ITGAV expression is negatively regulated by SIRT6**. *A*, E-box 1-Luciferase or E-box 2-Luciferase reporter was transfected into cells with expression of si-SIRT6. After 24 h, the luciferase activities were evaluated. *B*, A549 cells were transfected with si-SIRT6 for 24 h, then the mRNA levels of ITGAV and ICAM-1 were evaluated by qRT-PCR. *C*, cells were transfected with si-SIRT6 for 24 h, then the protein levels of ITGAV and ICAM-1 were evaluated by Western blot analysis. *D*, cells were transfected with si-si-GCIP or si-SIRT6 for 24 h before being assayed for ChIP with anti-c-Myc. PCR and qPCR (*E*) analyses were performed on the immunoprecipitated DNA samples using primers specific for E-box region in ITGAV gene promoter reported. *F*, cells were transfected with si-c-Myc, si-GCIP or si-SIRT6 for 24 h and then the cell extracts were pulled down by biotinylated double-stranded E-box oligonucleotides. DNA-bound SIRT6, c-Myc, and GCIP were collected with streptavidin-agarose beads for 1 h and evaluated by Western blot analysis. Data are representative of five to seven independent experiments.

Given that SIRT6 has been shown to interact with c-Myc and co-repress c-Myc-mediated gene expression (22), we questioned whether GCIP could coordinate with SIRT6 to associate with c-Myc on E-box regions and thereby suppress ITGAV expression. Indeed, the knockdown of SIRT6 enhanced the E-box-driven luciferase activities (Fig. 6*A*), mRNA levels (Fig. 6*B*), and protein levels (Fig. 6*C*) of ITGAV and ICAM-1. Consistent with the results of Fig. 5, ChIP-PCR and ChIP-qPCR analyses further indicated that the occupancy of c-Myc on the E-box was slightly attenuated by GCIP depletion and further decreased by SIRT6 depletion (Fig. 6, *D* and *E*). Using a biotinylated double-stranded oligonucleotide probe corresponding to E-box regions of the human ITGAV gene to precipitate bound proteins from cell extracts (oligonucleotide pull-down assay), we determined that GCIP and SIRT6 both specifically bind to a wildtype E-box probe in a c-Myc-dependent fashion (Fig. 6*F*). Consistent with the above-described PLA results, our oligonucleotide pull-down assay also showed that GCIP knockdown attenuated the occupancy of c-Myc on the E-box region and SIRT6 knockdown further lowered this occupancy. Together, our results indicate that GCIP interacts with SIRT6 and c-Myc in cells and contributes to regulating ITGAV gene expression. We further suggest that the formation of the GCIP-SIRT6-c-Myc complex might drive c-Myc to be tightly associated with an inhibitory region that is negatively regulated by c-Myc.

**Figure 7:**
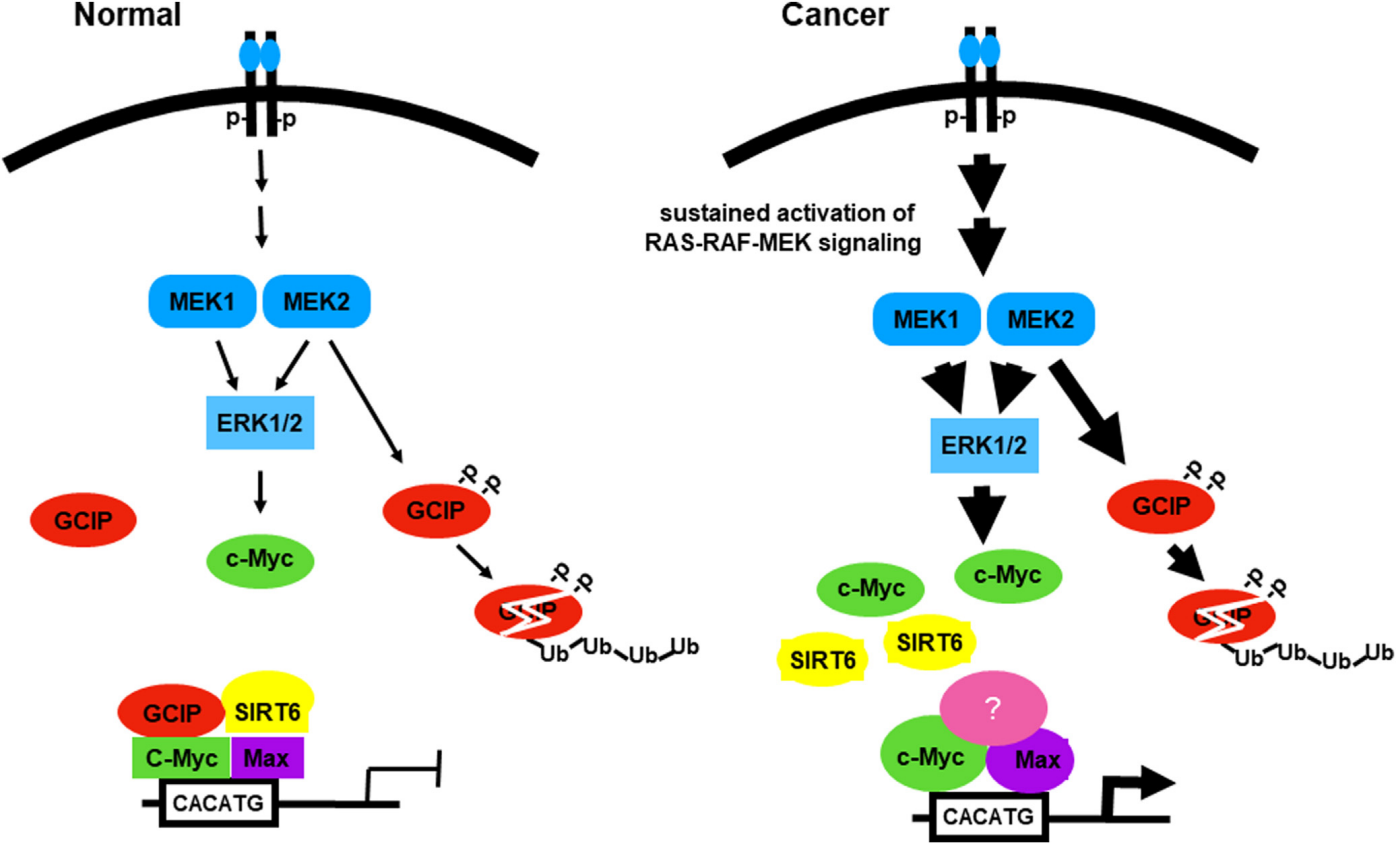
**Schematic representation of dynamic balance between GCIP-SIRT6 and MEK2-ERK-c-Myc signaling in the control of normal cell processes**. The study also suggests that there is a negative correlation between active MEK1/2-ERK and GCIP protein expression in cancer progression.

Mechanistically, our data reveal a regulatory network involving GCIP, SIRT6, c-Myc, and ITGAV and suggest that the SIRT6-GCIP complex possibly plays a role in negatively regulating the transcription activity of c-Myc on ITGAV expression (Fig. 7).

## Discussion

Our previous and present results provide a more comprehensive understanding of GCIP in tumorigenesis and identify a previously unrecognized role of GCIP: the ability to interact with c-Myc and recruit SIRT6 to E-box binding sites in the ITGAV promoter region. Using a combination of *in vitro* and *in vivo* studies, we herein reveal a novel mechanism in which GCIP interacts with the corepressor SIRT6 and c-Myc, binding to the E-box element of the ITGAV promoter to suppress its expression. Lorenzin *et al*. suggested that c-Myc regulates different sets of genes under physiological and oncogenic conditions due to differences in promoter affinities and concluded that such differences can underlie the specific gene expression profiles observed in biological systems with different c-Myc expression levels (48). This proposal is consistent with our results, suggesting that the biological functions of c-Myc under neoplastic conditions and the loss of GCIP expression support the progression of c-Myc-expressing tumors. Our results further show that GCIP is necessary for the c-Myc-mediated repression of ITGAV, establishing a possible signaling axis connecting GCIP, SIRT6, and c-Myc to explain the expression of integrin αv in tumorigenesis (Fig. 7).

Since c-Myc can activate or repress transcription, depending on the associated factors and the affinity of the target promoter, one cannot predict c-Myc-related gene regulation based on the presence or absence of its binding at a given locus (6, 48, 49). Most researchers believe that c-Myc interacts differentially with a variety of cofactors in dynamic and distinctly functional complexes to control target gene selection and downstream pathways. For instance, in the interaction of c-Myc with Miz-1 on the initiator element (Inr) of the p15 gene, c-Myc interferes with the recruitment of p300 to Miz-1 to abrogate Miz-1 function and suppress p15 expression (9). On the other hand, c-Myc repression of gene transcription requires a functional c-Myc/Max complex (50). In contrast, Hhex suppresses c-Myc-mediated gene expression and function through disruption of the c-Myc/Max complex (51). Recruitment of a corepressor to the c-Myc/Max complex also appears to be a critical mechanism for c-Myc-mediated transcriptional repression. The c-Myc-mediated repression of p21 transcription appears to involve the formation of a repressive complex that includes c-Myc, Miz-1, and DNA methyltransferase 3a (DNMT3a) (52). To suppress C/EBPδ expression, a repressive complex that includes c-Myc, Max, and Miz-1 associates with the C/EBPδ proximal promoter and the AAA + family DNA helicases, RuvBL1 and RuvBL2, interact with c-Myc to enhance its repression of C/EBPδ promoter activity (53). The histone methyltransferase, G9a, can interact with c-Myc as an epigenetic regulator of c-Myc/Max-mediated transcriptional repression (54). Although E-box motifs have been reported to strongly associate with c-Myc/Max-activated genes, Tu *et al*. reported genome-wide data showing that E-box and E-box variant motifs are strongly enriched in G9a-dependent sites, indicating that DNA sequence analysis alone is insufficient to identify c-Myc/G9a repressed genes (54). This implies that both epigenetic context and protein interactions crucially contribute to specifying c-Myc transcriptional activity. Therefore, the specific cofactors can act as molecular switches to mediate c-Myc transcriptional activity. C-Myc reportedly suppresses transcription from the promoters of the αv and β3 integrin genes (15), although the detailed mechanism underlying this remains to be explored. Cichon *et al*. reported that the TGF β-induced upregulation of integrin subunits requires the downregulation of c-Myc in MCF10 A basal breast cancer cells (55). In contrast, YY1 recruits c-Myc and HDACs to the α3 integrin promoter to downregulate its transcriptional activity (11). Our present results demonstrate that GCIP interacts with c-Myc to repress transcription from the promoter of the αv integrin gene (Fig. 7). SIRT6 was previously reported to physically interact with RelA/p65 to attenuate the expression of NF-kB target genes, including ICAM-1 (21). From this and our present findings, we hypothesize that GCIP coordinates with SIRT6 to associate with c-Myc to decrease acetylation around the promoter regions of ITGAV and ICAM-1 and thereby suppress their gene expression. Our PLA results indicated that Max and GCIP were found in close proximity to one another, supporting their potential interaction (Fig. 2*D*). Notably, the knockdown of Max significantly attenuated the interaction of GCIP and c-Myc (Fig. 2*E*). Given this, and in light of the coalition model (56), we cannot exclude the possibility that Max participates in the GCIP-SIRT6-c-Myc complex-mediated repression of ITGAV expression.

Mechanistically, GCIP is considered to act as a transcriptional repressor through diverse mechanisms that determine the transcriptional repression effects. Such effects could include the recruitment of corepressors to sterically obstruct transcription factor binding to the TATA box, the recruitment of chromatin modifiers to transcription factor-binding regions to alter the epigenetic landscape, or direct interaction with other transcription factors to hinder transcription factor function. Therefore, understanding the interplay between GCIP-mediated negative regulation of cell proliferation and tumorigenesis and GCIP-mediated transcriptional regulation will critically support efforts to understand tumor progression and develop more effective treatments. Ikushima *et al*. previously reported that GCIP physically interacts with oligodendrocyte transcription factor 1 (Oligo 1, a class-II bHLH transcription factor) to disrupt its TGF-β signaling-dependent association with Smad2/3 and that overexpression of GCIP suppresses TGF-β-induced cell growth and cell migration in glioma (40). Fujita *et al*. described a novel mechanism wherein GCIP directly interacts with CBP to inhibit the interaction between CBP and RNA polymerase II and thus suppress CREB- and NF-κB-dependent transcription (42). In this study, we identify GCIP as a novel binding protein of c-Myc and propose a unique mechanism wherein GCIP interacts with SIRT6 to bind c-Myc on the E-box of the ITGAV promoter and thereby repress ITGAV expression. SIRT6 has been previously reported to interact with c-Myc and co-repress c-Myc-mediated gene expression (22). We found that the interaction of SIRT6 with c-Myc was slightly decreased by GCIP knockdown and the interaction of c-Myc with GCIP is at least partly dependent on the presence of SIRT6, as shown by coimmunoprecipitation (Fig. 5, *A* and *B*), PLA (Fig. 5, *C* and *D*), ChIP-PCR (Fig. 6, *D* and *E*), and oligonucleotide pull-down (Fig. 6*F*) analyses. These data might indicate that there is a cooperative interaction between GCIP/SIRT6 and the c-Myc complex. Moreover, our *in vitro* pull-down assays suggested that there are interactions between GCIP, SIRT6, and the c-Myc complex (Fig. 4*C*). In sum, our present findings and the previous reports together reveal multiple mechanisms that contribute to the GCIP-mediated suppression of tumor growth and migration.

c-Myc is dysregulated in many human tumors, often exhibiting constitutive and elevated expression. A wide variety of mechanisms contribute to the dysregulation of c-Myc. Among them, RAS-RAF-ERK signaling, which is often activated in cancers, has been intensively investigated for its critical roles in regulating c-Myc expression at the transcriptional and post-translational levels, such as by elevating c-Myc mRNA expression and phosphorylating c-Myc at Ser62 to prevent its proteasomal degradation (57). Many tumors exhibit upregulation of RAS-RAF-ERK signaling, and over 30% of tumors bear upregulated c-Myc expression. We previously identified GCIP as a new physiological substrate for MEK2, but not MEK1, showed that MEK2 directly phosphorylates GCIP at its Ser313 and Ser356 residues, and reported that this enhances proteasomal degradation to promote tumor cell proliferation and migration (46). Our previous and present findings uncover a new function for RAS-MEK signaling in controlling cell proliferation and suggest that the regulation of MEK2-GCIP may provide a dynamic physical regulatory loop between proliferation and growth suppression. It is likely that MEK concurrently governs ERK signaling to activate c-Myc and growth-related effectors while also antagonizing the suppressive effect of GCIP by promoting its proteolysis.

Integrin αv (also called CD51, encoded by ITGAV) is a transmembrane glycoprotein that is responsible for the adhesion of cells to extracellular matrix components, such as fibronectin and vitronectin. Integrin complexes also directly participate in cell proliferation and survival. Overexpression of integrin αv has been reported in many human cancers (58) and appears to be involved in the tumor metastasis of melanoma, prostate, breast (59), brain (60), and colorectal (61) cancer. Higher levels of integrin αv have been associated with advanced malignancy in colorectal cancer (62). Here, we propose a novel mechanism in cancer, wherein elevated MEK/ERK signaling leads to GCIP down-regulation (46) and thereby enhances ITGAV expression and cell migration. We also provide significant evidence supporting the idea that GCIP exerts a tumor suppressor function in decreasing cancer cell migration and metastasis.

Advances in understanding molecular aberrations in malignant cancers have led to the development of efficient targeted therapies. We herein show mechanistically that GCIP and SIRT6 work together to modulate c-Myc at the promoter region of the ITGAV gene and thereby dampen ITGAV expression. Moreover, cancer cell behavior is remarkably malleable when the level of GCIP is modulated: GCIP expression can potentially reduce metastatic properties, such as MEK-ERK signaling, whereas its knockdown may augment these properties.

## Experimental procedures

### Cell culture

The A549, CL1-0, CL1-5, AGS, and MKN-45 cell lines were as previously described (46). Fetal bovine serum (FBS) and penicillin/streptomycin were obtained from Invitrogen. All cells were cultured in RPMI 1640 medium (Invitrogen) supplemented with 10% FBS, at 37 °C in a humidified incubator containing 5% CO_2_ in air.

### Chemicals and antibodies

The antibody against His-Tag was purchased from Millipore. The protein G sepharose, glutathione sepharose 4B, and Nickle Sepharose 6 Fast Flow were obtained from GE Healthcare. Antibodies specific for GCIP, ITGAV, and ICAM-1 were from BD Bioscience. Antibodies specific for SIRT6 and c-Myc were purchased from Cell Signaling Technology. The antibody against beta-actin was purchased from Santa Cruz Biotechnology Inc. The antibody against Max was from Atlas antibodies (Bromma). The peroxidase-conjugated secondary antibodies against mouse and rabbit IgG were from Jackson ImmunoResearch Laboratories. The Jetpei transfection reagent was from Polyplus. Double-stranded RNA duplexes targeting GCIP, c-Myc, Max, and SIRT6, along with a non-targeting siRNA, were purchased from ON-TARGET plus SMARTpool (Dharmacon Research). Cells were transfected with siRNA using Lipofectamine RNAiMAX (Invitrogen) in glucose-free Opti-MEM (Invitrogen) according to the manufacturer’s recommendations. All other chemicals were purchased from Sigma-Aldrich or Amresco.

### cDNA microarray

Changes in gene expression in response to GCIP knockdown were evaluated in A549 cells. Briefly, A549 cells were transfected for 24 h with si-GCIP or control siRNA and total RNA was isolated. cDNA microarray analysis was conducted by the Phalanx Biotech Group using a system of 32,050 60-mer sense-strand oligonucleotides that included 30,968 human genome probes and 1082 experimental control probes. The transcripts differentially expressed (*p* < 0.05) in si-GCIP cells compared to si-control cells were evaluated. The microarray analysis was performed two times in duplicate.

### Quantitative real-time PCR (qRT-PCR)

Total RNA extraction, specific primer designation, and quantitative real-time PCR analysis were performed as previously described (46). Each sample was analyzed three times in triplicate. HPRT (hypoxanthine phosphoribosyltransferase) was used as the loading control. Fold changes in mRNA expression between cells were determined by 2^–ΔΔCT^ normalization.

### Transwell migration and invasion assay

Transwell migration assay was performed as described previously (46). To assess *in vitro* invasion, the assay was performed using transwell devices loaded with 1 mg/ml basement-membrane matrigel (Millipore) according to the manufacturer’s recommendations. Briefly, 1 x 10^4^ cells were plated in the top compartment of each well in a serum-free medium and allowed to invade through the Matrigel into a medium containing 10% FBS for 24 h. The non-invaded cells in the top compartment of each well were scraped off, and the invaded cells were fixed in methanol and stained with 1% crystal violet in 75% ethanol. In both cases, the cell numbers were counted and quantified under microscopy. The images shown are representative of at least three independent experiments carried out under the same conditions.

### Proximity ligation assay

Proximity ligation assay (PLA) was performed using the Duolink PLA mouse/rabbit kit (Sigma) according to the manufacturer’s instructions and as described in our previous study (63, 64). The primary antibodies used were mouse anti-GCIP (1:50, Millipore), rabbit anti-c-Myc (1:200, Millipore), or rabbit anti-SIRT6 (1:200, Cell signaling). The same analytic parameters were used consistently throughout all experiments. The images shown are representative of at least three independent experiments carried out under the same conditions. The quantification of fluorescence density was determined by ImageJ.

### Luciferase reporter assay

Two sets of oligomers, one containing E-box 1 and one containing E-box 2 of the *ITGAV* promoter region (15), were synthesized and cloned into the *Kpn*I and *Xho*I sites of the pGL3-promoter vector (Promega, Madison, Wisconsin, USA). The sequence of mutant E-Box 1 is "ACACGT", and the sequence of mutant E-Box 2 is "ACAAGT". Plasmids were transfected into cells using Jetpei (Polyplus, Illkirch, France) according to the manufacturer's recommendations. After 24 h, cell extracts were prepared and a Bright-Glo Luciferase Assay System (Promega) was used according to the manufacturer's instructions. Luminescence was measured using a Sirius Luminometer (Berthold Detection System). All samples were assayed in triplicate.

### Chromatin immunoprecipitation (ChIP)-PCR/qPCR assay

Cells were transfected with GCIP-His or si-c-c-Myc and ChIP was performed using an EZ-Magna ChIP kit (Millipore), as recommended by the manufacturer. Briefly, cells were cross-linked with 1% formaldehyde. Chromatin was fragmented with a Sonics Vibra-cell system (Sonics & Materials, Inc.) and subsequently immunoprecipitated with anti-c-Myc, anti-His-tag, anti-RNA polymerase II (positive control antibody), or non-specific IgG antibodies (negative control antibody). After purification, the immunoprecipitated DNA was amplified by PCR using primers specific to the E-box region in the *ITGAV* promoter reported by Liu *et al*. (15) and resolved on 3% agarose DNA gels. In addition, the immunoprecipitated DNA was further analyzed by qPCR using the SYBR Green method. For the positive control experiment, immunoprecipitation of anti-RNA polymerase II-associated DNA fragments was verified by PCR using primers against the *GAPDH* promoter region, as recommended by the manufacturer (Millipore). The density of PCR products was determined by ImageJ.

### Biotinylated oligonucleotide precipitation assays

Cells were lysed in a lysis buffer containing protease inhibitors. Cellular debris was removed by centrifugation, and 2 mg cell extract was mixed with 1 μg of biotinylated double-stranded oligonucleotides and streptavidin-agarose beads (Pierce) overnight at 4 °C. DNA-bound proteins were collected by centrifugation, washed with lysis buffer, separated on an SDS-polyacrylamide gel, and identified by Western blotting (65). Quantification of Western blot was performed by ImageJ.

### Co-immunoprecipitation

Cells were harvested and lysed in lysis buffer with protease inhibitors. After centrifugation, the supernatants were collected. For each immunoprecipitation reaction, 2 mg cell extract was mixed with 1 μg of a specific antibody and protein G agarose beads, or the cell lysate was mixed with IgG alone, and inverted at 4 °C overnight. Beads were washed three times with cold lysis buffer and then resuspended in lysis buffer with protease inhibitors. Immunoprecipitated proteins were eluted by boiling at 95 °C for 5 min and analyzed by Western blot. Quantification of Western blot was performed by ImageJ.

### Statistical analysis

For statistical analysis, each experimental value was compared to its corresponding control. The results from at least three independent experiments are presented as the mean ± standard deviation. The statistical significance of differences between mean values was estimated using the *t* test. *p < 0*.*05* was considered statistically significant.

## Data availability

All data described in this study are included in the main article.

## Supporting information

This article contains supporting information.

## Conflicts of interest

The authors declare that they have no conflicts of interest with the contents of this article.
