# Supplementary material for: GCIP and SIRT6 cooperatively suppress ITGAV gene expression by modulating c-myc transcription ability
Source: J Biol Chem. 2025 Feb 13;301(3):108314. doi: 10.1016/j.jbc.2025.108314 (PMC11930424; doi:10.1016/j.jbc.2025.108314)

**Supporting information**

**GCIP and SIRT6 Cooperatively Suppress *ITGAV* Gene Expression by Modulating c-Myc Transcription Ability**

Yi-Ching Huang^1^, Tien-Ming Yuan^2,3^, Bang-Hung Liu^1^, Ruei-Yue Liang^1^, Kai-Li Liu^4,5^, and Show-Mei Chuang^1,6^**^*^**

**S1** Raw data of cDNA microarray (excel file).

**S2** Microarray analysis of si-GCIP in A549 cell line. (A) Volcano plot of cDNA microarray analysis. Plots showed differences between the lack of GCIP and the control. Differentially expressed cDNA with fold change≧2 and P <0.05 are shown in red. (B) KEGG analysis of si-GCIP/si-Control (fold change≧2, P value<0.05).

**S2A**


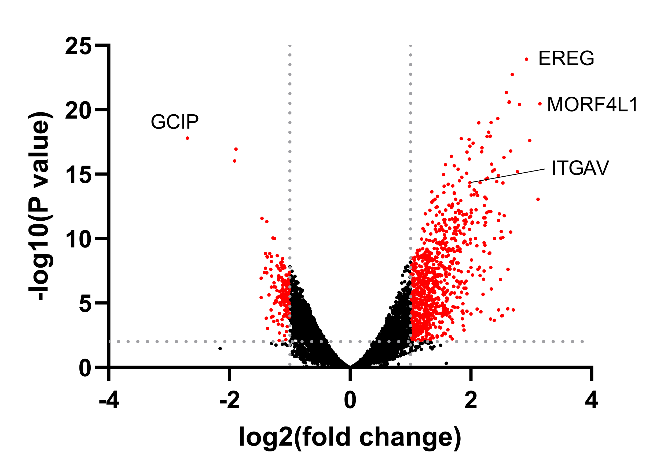


**S2B**


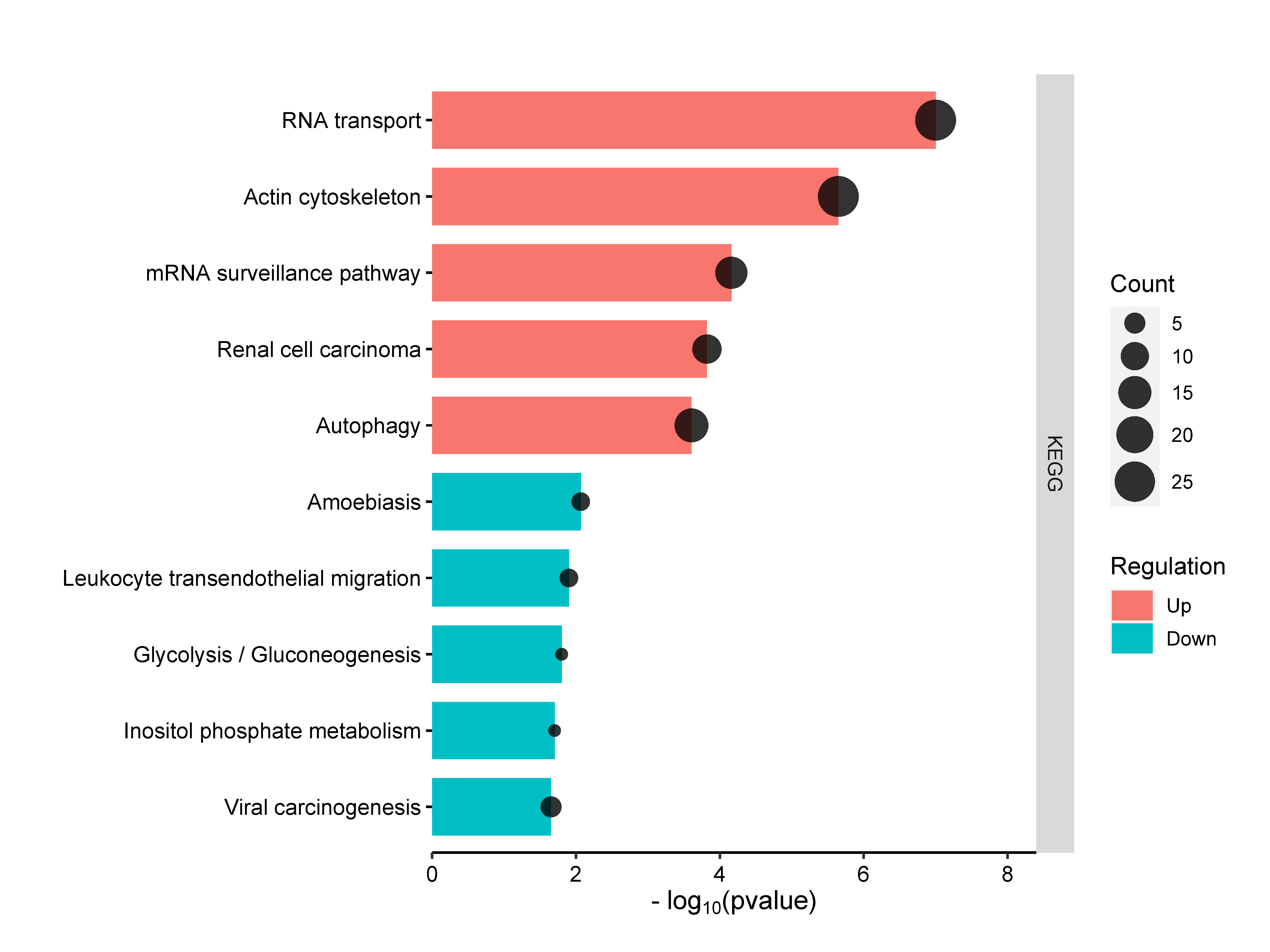

Supplement: Supporting information 2 [file mmc2.docx]
